# High plasma soluble levels of the immune checkpoint HLA-G molecule among bodybuilders

**DOI:** 10.1371/journal.pone.0238044

**Published:** 2020-09-30

**Authors:** Talita M. Fernandes, Enrico F. Puggina, Celso T. Mendes-Junior, Milena C. de Paula, Paulin Sonon, Eduardo A. Donadi, Ana Paula M. Fernandes

**Affiliations:** 1 School of Physical Education and Sport of Ribeirão Preto, University of São Paulo at Ribeirão Preto, Ribeirão Preto, SP, Brazil; 2 Ribeirão Preto Faculty of Philosophy, Sciences and Letters, University of São Paulo at Ribeirão Preto, Ribeirão Preto, SP, Brazil; 3 College of Nursing, General and Specialized Nursing Department, University of São Paulo at Ribeirão Preto, Ribeirão Preto, SP, Brazil; 4 FIOCRUZ Oswaldo Cruz Foundation–Instituto Aggeu Magalhães, Federal University of Pernambuco, Recife, Brazil; 5 Medical School, Department of Medicine, University of São Paulo at Ribeirão Preto, Ribeirão Preto, SP, Brazil; San Raffaele Roma Open University, ITALY

## Abstract

**Introduction:**

Studies report that intense physical activity influences the down-regulation of immune function in athletes as well as the interaction between adipose tissue and the immune system.

**Aim:**

This study aimed to compare the plasma soluble levels of the immune checkpoint HLA-G (sHLA-G) molecule with the fat mass and muscle mass index among 77 bodybuilders and 64 controls.

**Results:**

The comparisons of the percentage of body fat (%BF) revealed that the groups of male and female bodybuilders showed a statistically significant reduction in the percentage of body fat when compared to their control group, (P <0.0001, for both comparisons). Regarding sHLA-G levels, the comparisons showed that the group of male bodybuilders had significantly higher sHLA-G levels compared to the group of female bodybuilders (P = 0.0011).

**Conclusion:**

Our results showed that in bodybuilders with less body fat, the systemic levels of soluble HLA-G, an immunological molecule with recognized immunosuppressive function, are significantly higher and suggest that this immune mechanism may corroborate the immunosuppressive state in athletes undergoing intense and prolonged physical training.

## Introduction

In contrast to moderate or intermittent physical activity, prolonged and intensive exertion causes numerous changes in immunity that possibly reflects on immunological suppression. In fact, evidence indicates that intense physical activity may downregulate the immune function and increase the risk of certain types of infection [1, 2]. Indeed, bodybuilding athletes are submitted to high intensity resistance training to develop muscular hypertrophy (size), to reduce the amount of subcutaneous fat, and to slow down the rate of recovery from fatigue after exercise [3–5].

Exercise-induced immune depression has a multifactorial origin, depending on mechanisms related to neuro/immune/endocrine systems. Evidence shows that prolonged periods of intense training may alter the profile of immune cells, including lymphopenia [6, 7] mucosal immunoglobulin levels [3], impaired phagocytosis [8, 9], and natural killer cell cytotoxicity (NKCA) [10].

Human leukocyte antigens (HLA) are involved in several important functions of the immune system, starting from antigen presentation to lymphocytes, performed by the classical class I histocompatibility molecules (HLA-A/B/C), and extending until the control of the immune response, as performed by the non-classical class I molecules (HLA-E/F/G). Among these molecules, HLA-G is the most studied one, and its major function is the down-regulation of the activity of the innate and adaptive immune system cells, by means of the interaction with ILT-2/4 inhibitory leukocyte receptors. Accordingly, HLA-G may inhibit the proliferation of T and B lymphocytes [11, 12], the activity of antigen-presenting cells (APC) [13] and the cytotoxicity of TCD8 and Natural Killer (NK) cells [11]. Because of these properties, HLA-G has been recognized as an immune check point molecule. In certain infections and in some types of cancer, the overexpression of HLA-G can create a tolerogenic environment; inhibiting several steps of the immune response, propitiating the spreading of infectious and malignant cells [14]. In contrast, in transplanted organs and autoimmune disorders the expression of HLA-G may produce beneficial effects.

Considering that: i) numerous studies report that intense physical activity may down-regulate the immune function in athletes [5, 8–10], ii) the reported interactions between adipose tissue and the immune system [15] may be affected by the low amount of subcutaneous fat in bodybuilders [3, 4], and iii) HLA-G down-regulates the function of cells of the innate and adaptive immune system [11–14], this study aimed to evaluate the soluble HLA-G levels among bodybuilders, stratified according to fat mass and muscle mass index.

## Materials and methods

### Sample

The sample consisted of 141 healthy individuals categorized into 77 bodybuilders and 64 controls, who presented no infections, immunological or metabolic disorders at the time of inclusion in the study. The bodybuilder group was composed of 50 (65%) male bodybuilders (MB) with a mean age of 33.6 years and 27 (35%) female bodybuilders (FB) with a mean age of 33.2 years. The control group was composed of 24 (37.5%) female controls (FC) with a mean age of 28.0 years and 40 (62.5%) male controls (MC) with a mean age of 23.5 years. Due to lack of data for all individuals, analyses related to height, total body weight, lean, fat and body mass indexes were performed only with 14 females and 31 males in the control and 19 females and 33 males in the bodybuilders. The group of high performance bodybuilders was selected from the participants of the 48^th^ Brazilian Championship of Bodybuilding and Fitness–IFBB 2018, held in the city of Ribeirão Preto, SP, Brazil. The athletes were assessed before or after the standard weighing process to championship. All bodybuilders reported following the American College of Sports Medicine (ACSM) recommendations for training with respect to hypertrophy. Bodybuilders preparing for competition followed self- or coach- prescribed diets, with the sole aim of supplying specific amounts of protein, fat and carbohydrate. The control group was selected from bodybuilders with at least 1 year of practicing the sport. This investigation was approved by the Ethics in Research Committee of the University of São Paulo at Ribeirão Preto School of Physical Education and Sport of Ribeirão Preto (EEFERP-USP, process#2,808,296). The athletes agreed to take part in this investigation by signing an informed consent form.

### Anthropometry

Measurements were taken according to the International Society for the Advancement of Kineanthropometry guidelines (ISAK) [16], and from each subject the following variables were taken: i) anthropometric assessment for the determination of body composition (% of fat, lean mass, fat mass and BMI—Body Mass Index), ii) total weight, total stature, nine skinfolds (tricipital, subscapular, bicipital, chest, medium axillary, suprailiac, abdominal, front thigh, and medium calf), two muscle girths (flexed biceps, calf standing), and three bone breadths (elbow, ankle and knee).

The following evaluations were taken: i) weight was measured using the *Welmy*® (Santa Barbara D´Oeste, BRA) scale, with accuracy of 100 g; ii) height with a vertical metric scale with a 1 mm accuracy; iii) skinfold thickness was assess by a adipometer *Cescorf*® with a 0.2 mm accuracy; and iv) muscle girths were measured with a measuring tape *Sanny Medical* (Guangdong, China) with a 1 mm accuracy scale. After obtaining these values, we used the following equation to estimate body density of male athletes: DC (g/cm^2^) = 1,112–0.00043499 (∑7doc) + 0.00000055 (∑7doc)^2^- 0.00028826 (age); and, for women: DC (g/cm^2^) = 1.0970 - [0.00046971 (ST) + 0.00000056 (ST)^2^]—[0.00012828 (age)], developed by Jackson and Pollock, 1978 [17], followed by converting the result into a percentage of fat by applying the Siri Equation (1961):% G = [(4.95/D) - 4.50] x 100 [18].

### Soluble HLA-G quantification

Soluble HLA-G (sHLA-G) was quantified using MEM-G/9 antibody, which recognizes the most abundant soluble isoforms (shed sHLA-G1 and secreted HLA-G5), and an anti-human β2-microglobulin antibody, respectively, as capture and detection antibodies [19]. Microtitration plates were coated with 10 μg/mL MEM-G/9 mouse-anti-human HLA-G mAb (Exbio, Praha, Czech Republic) and incubated overnight at 4°C. Plates were saturated with 300 μL ready to use diluent buffer (DAKO, Carpinteria, CA, USA) for 2 h. Plasma samples were diluted (½) in diluent buffer, tested in duplicate and incubated for 2 h. After incubation and washing, monoclonal anti-human beta2-microglobulin antibody (DAKO, Glostrup, Denmark), which recognizes the immobilized antibody sHLA-G complex, was added to the wells and incubated for 1 h. The plates were then incubated for 1 h with 100 μL (1:200) envision buffer + system HRP (DAKO, Carpinteria, CA, USA) to obtain anti-β2-microglobulin-horseradish peroxidase complex to improve the efficiency of the reaction. All incubation steps were performed at room temperature and followed by four washes using washing buffer (H_2_O, PBS 1X, 0.1% Tween 20). The plates were incubated for 30 min with substrate (Tetramethylbenzidine super sensitive-TMB, Sigma Aldrich, Saint Louis, MO, USA) and absorbance was measured at 450 nm after adding HCL (1 N). Total sHLA-G levels were determined from a five-point standard calibration curve using dilutions (12.5–200 ng/mL) of HLA-G5 purified from M8-HLA-G5 cell line culture supernatants. Results are presented in ng/mL.

### Statistical analysis

Categorical variables were described using proportions and 95% confidence interval, and the continuous variables were described by the mean, median, amplitude and correlation. Continuous variables were compared by means of Student T test, Wilcoxon-Mann-Whitney rank-sum test and analysis of variance (ANOVA). We checked for normality of distribution using Kolmogorov-Smirnov normality test. Since deviations from normality were found, non-parametric tests were used, Kruskal-Wallis and Mann-Whitney tests. In all analyzes performed, two-tailed versions of the tests were used and a significance level of 5% (alpha = 0.05) was adopted. Such analyzes were performed using the program GraphPad InStat version 3.06, GraphPad Software (www.graphpad.com). For intergroup analyzes, Spearman's correlation was used, an analysis used to discover the relationship between the two variables that do not have a normal joint distribution.

## Results

### Anthropometric assessment

Total body mass (body weight) and height were evaluated, and the body weight for the MB group ranged from 67–108 kg (84.97 ± 10.45), for the FB group it ranged from 45.5–77 kg (58.45 ± 9.56), for MC it ranged from 59–98 kg (79.17 ± 11.64) and FC of 48–74 kg (642.42 ± 8.80). The height of individuals in the MB group ranged from 160–186 cm (175.63 ± 6.29), for the FB group from 153–176 cm (164.68 ± 6.94), for MC it ranged from 164–192 cm (176.10 ± 6.80) and FC of 153–170 cm (162.18 ± 6.151) (Table 1).

**Table 1:** Anthropometric variables and comparison of the groups of individuals studied.

							Statistical Comparisons (Mann-Whitney Test)				
Variables	FC (*n* = 24)	MC (*n* = 40)	FB (*n* = 27)	MB (n = 50)	TC (*n* = 64)	TB (*n* = 77)	FC vs MC	FC vs FB	MC vs MB	FB vs MB	TC vs TB
	Mean (SD)	Mean (SD)	Mean (SD)	Mean (SD)	Mean (SD)	Mean (SD)					
	Median	Median	Median	Median	Median	Median					
	Min—max	Min—max	Min—max	Min—max	Min—max	Min—max					
**Age (years)**	29.042 (9.134)	24.325 (6.391)	33.222 (5.807)	33.420 (8.732)	26.094 (7.813)	33.351 (7.791)	**0.0321**	**0.0416**	**< 0.0001**	0.5861	**<0.0001**
	27.500	22.500	33.000	31.500	24.000	32.000					
	16–48	18–44	24–45	20–57	16–48	20–57					
Height (cm)*	162.18 (6.151)	176.10 (6.808)	164.68 (6.945)	175.63 (6.291)	171.77 (9.234)	171.63 (8.378)	**< 0.0001**	0.3579	> 0.9999	**< 0.0001**	0.9222
	165	175	164	176	172	173					
	153–170	164–192	153–176	160–186	153–192	153–186					
Total body mass (kg)*	62.429 (8.806)	79.173 (11.641)	58.453 (9.561)	84.971 (10.454)	73.963 (13.295)	75.282 (16.343)	**0.0002**	0.2258	0.0729	**< 0.0001**	0.5850
	66.700	77.600	56.000	83.000	74.000	77.500					
	48–74	59–98	45.5–77	67–108	48–98	45.5–108					
BMI (Kg/m^2^)*	23.685 (2.773)	25.448 (2.854)	23.014 (3.196)	27.526 (2.959)	24.900 (2.917)	25.305–4.033	0.0931	**0.0213**	**0.0156**	**< 0.0001**	0.7106
	24.250	25.230	22.310	26.570	24.540	25.715					
	19.540–29.900	20.720–31.770	18.000–26.040	22.340–35.270	19.540–31.770	18.000–35.270					
**%BF**	28.741 (8.766)	16.715 (6.438)	10.593 (2.798)	6.460 (5.183)	21.225 (9.389)	7.909 (4.893)	**< 0.0001**	**< 0.0001**	**< 0.0001**	**< 0.0001**	**< 0.0001**
	28.335	16.620	10.560	5.055	20.320	6.800					
	3.030–42.610	5.560–31.790	4.220–17.810	0.4500–29.950	3.030–42.610	0.4500–29.950					
LMI (kg)*	44.471 (5.015)	65.170 (7.824)	52.461 (8.572)	78.555 (10.493)	58.731 (11.962)	69.021 (2.219)	**< 0.0001**	**0.0062**	**< 0.0001**	**< 0.0001**	**0.0008**
	44.185	66.070	49.540	76.630	59.660	70.385					
	36.270–54.650	49.300–82.150	41.170–70.680	56.740–102.87	36.270–82.150	41.170–102.87					
FMI (kg)*	17.957 (5.876)	14.002 (7.459)	5.992 (2.029)	6.416 (5.534)	15.233 (7.181)	6.261 (4.551)	**0.0485**	**< 0.0001**	**< 0.0001**	0.1514	**< 0.0001**
	18.800	13.660	5.890	4.910	13.970	5.045					
	9.320–28.410	3.940–31.150	2.700–10.710	2.220–27.010	3.940–31.150	2.220–27.010					
**sHLA-G (ng/mL)**	86.549 (87.606)	166.42 (56.300)	76.518 (61.300)	129.17 (135.29)	136.47 (287.76)	110.70 (117.16)	0.0856	0.7989	0.2574	**0.0011**	0.3767
	60.000	90.000	67.270	109.29	78.335	99.290					
	0.000–355.45	4.580–2266.4	8.890–330.00	0.000–932.14	0.000–2266.4	0.000–932.14					

Statistical significant values are highlighted in boldface.

*Sample sizes for these variables are reduced. For FC, *n* = 14; for MC, *n* = 31; for FB, *n* = 19; for MB, *n* = 33; for TC, *n* = 45; and for TB, *n* = 52.

BMI = body mass index (median); FC = female control; MC = male control; FB = female bodybuilder; MB = male bodybuilder; vs = versus.

Comparisons of the Body Mass Index (BMI) between the different groups showed that the group of Male Bodybuilders has a significantly higher BMI compared to the Male Control (P = 0.0213) and Female Bodybuilders group (P <0.0001) (Table 1).

### Percentage of Body Fat (%BF)

Comparisons of the percentage of body fat (%BF) between the groups revealed that the groups of male and female bodybuilders showed a statistically significant reduction in the percentage of body fat when compared to their control group, male (P <0.0001) and female (P <0.0001), respectively. The comparison between the groups of male and female bodybuilders showed that the group of male bodybuilders showed a significantly greater reduction in the percentage of body fat compared to the group of female bodybuilders (P <0.0001) (Table 1).

### Lean Mass Index (LMI)

To enhance fitness in bodybuilders, LMI must have high levels associated with low levels of fat mass index. The LMI was calculated using the formula LMI = TBM-FMI, where: LMI = Lean Mass Index, TBM = Total Body Mass, FMI = Fat Mass Index. Table 1 shows the medians and comparisons between the different groups studied.

Comparisons of the LMI between the groups showed that the groups of male and female bodybuilders have significantly higher LMI compared to the male (P<0.0001) and female (P = 0.0062) control groups, respectively. The control groups and male bodybuilders had significantly higher LMI compared to the control (P<0.0001) and female bodybuilder (P<0.0001) groups, respectively.

### Fat Mass Index (FMI)

For the calculation of fat mass, the formula FMI = TBM x %BF/100 was used, where: FMI = Fat Mass Index, TBM = Total Body Mass, %BF = Body Fat Percentage. Table 1 shows the medians and comparisons between the different groups studied.

Comparisons of the FMI between the groups showed that male and female bodybuilders have significantly lower FMI compared to the male (P <0.0001) and female (P <0.0001) control groups, respectively. Among the controls, the male control group had significantly lower FMI compared to the female control group (P = 0.0485).

### Plasma levels of soluble HLA-G

**Fig 1:**
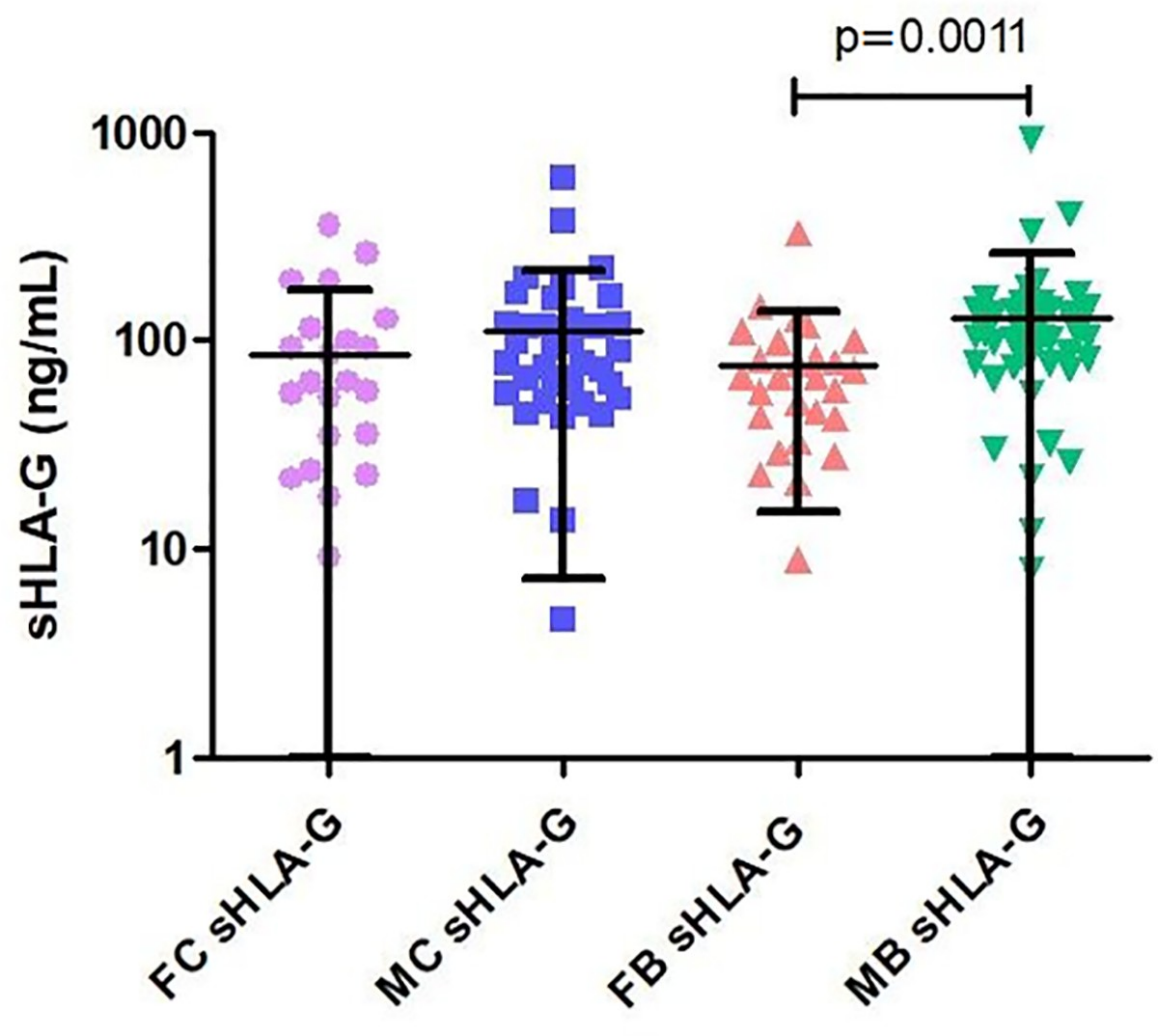
Plasma levels of soluble HLA-G (sHLA-G) from individuals in the FC (female control), MC (male control), FB (female bodybuilder), MB (male bodybuilder) groups. sHLA-G levels among MB were significantly higher when compared to FB. The horizontal bars in bold represent the medians.

Plasma levels of soluble HLA-G were analyzed and for the FM group their levels ranged from 0–932.14 ng/mL (109.29 ± 135.29), for the FF group from 8.89–330 ng/mL (67.27 ± 31.3), for CM of 4.58–604.58 ng/mL (88.75 ± 105.37) and CF of 0–355.45 ng/mL (60 ± 87.6) (Fig 1).

Comparisons of plasma levels of soluble HLA-G (sHLA-G) showed that the group of male bodybuilders had significantly higher levels of sHLA-G compared to the group of female bodybuilders (P = 0.0011) (Table 1).

Considering that the age difference between the groups MB (33.6 years) and MC (23.5 years) was of almost 10 years, we also correlated results according to age and gender in the four studied groups. According to age, the following correlations were performed: i) (MB: rho = 0.0233; P = 0.8724; ii) FB: rho = -0.0724; P = 0.7196; iii) MC: rho = -0.0330; P = 0.8400; and FC: rho = 0.2936; P = 0.1638). According to gender, the following correlations were evaluated: i) (males: rho = 0.1032; P = 0.3330; females: rho = 0.1708; P = 0.2307) or in the entire sample (rho = 0.0872; P = 0.3037). These analyses revealed that age and gender did not influence intragroup results.

When compared to the different groups, male bodybuilders (MB) had significantly higher values of sHLA-G and significantly lower values of Fat Mass Index (FMI) and Percentage of Body Fat (%BF), we explored intra-group comparisons (Table 1).

Intragroup correlations (Table 2) showed that MB individuals with lower %BF had significantly higher levels of sHLA-G (P = 0.0073). Similarly, individuals with lower FMI had significantly higher levels of sHLA-G (P = 0.0087).

**Table 2:** Spearman correlations between plasma levels of soluble HLA-G with Body Fat Percentage, Lean Mass Index and Fat Mass Index among bodybuilders.

sHLA-G (ng/mL)	%BF	LMI (kg)	FMI (kg)
**Female (*n* = 27)**^**a**^	*p* = 0.1689	*p* = 0.8194	*p* = 0.1827
**Male (*n* = 50)**^**b**^	*p* = **0.0073**	*p* = 0.8224	*p* = **0.0087**

Significant values are highlighted in boldface.

^a^ For correlation with LMI and FMI, a reduced sample size of 19 women was used.

^b^ For correlation with LMI and FMI, a reduced sample size of 33 men was used.

## Discussion

The immune system presents several mechanisms for regulating the inflammatory response. Several immunological mediators have anti-inflammatory and immunoinhibitory functions, among these, HLA-G presents a well-recognized immunomodulatory activity [17–20]. This is the first study to assess plasmas HLA-G levels in bodybuilders, highlighting male to female and intragroup differences. In short, sHLA-G levels were augmented in: i) male bodybuilders (MB) compared to female bodybuilders (FB) (P = 0.0011), ii) the correlation between MB evidenced an inverse relationship between body fat and sHLA-G levels.

Studies have shown that adipose tissue secretes various inflammation-related cytokines, such as leptin and IL—6, which can induce chronic low-grade inflammation in obese individuals, with a concomitant stimulation of sHLA-G production, which has anti-inflammatory activities [21, 22]. This mechanism is possibly due to compensate the chronic pro-inflammatory state of obesity. Other studies indicate that the presence of higher levels of sHLA-G in patients with chronic diseases could, provisionally, represent a way to compensate the inflammatory condition [23].

Studies that measured the chronic effects of moderate or intermittent physical exercise point to local and systemic reduction of pro-inflammatory mediators, with attenuation in the production and secretion of acute phase proteins [24], and greater production and secretion of cytokines with anti-inflammatory function (especially IL-6 in skeletal muscle tissue and blood) [25]. The literature also suggests that the beneficial effects of physical training on the modulation of inflammation depend on the quality and quantity of stimuli, which are directly related to the rest time between stimuli to avoid the emergence of the stress condition [26]. In this context, it is also reported that physical exercise when intensively performed contributed to increase the incidence of upper respiratory tract infection, while exercise of moderate intensity would have a protective effect against the risk of infections [27, 28].

Considering the relationship between susceptibility to infections and inflammatory\immunologicalmediators associated with the practice of physical exercise, we sought to understand what possible mechanisms may modulate the immune response, primarily focusing on HLA-G. According to Costa Rosa and Vaisberge, 2002 [26], cellular and humoral components are mobilized from the immune system in response to mechanical (hypoxia, hyperthermia and muscle damage), metabolic (glutamine) and hormonal (adrenaline, cortisol) changes imposed by exercise [27].

In normal muscle, HLA-G is undetectable. However, in the context of inflammation, muscle fibers express HLA-G in a distribution similar to classical HLA class I molecules, as well as under stimulation of inflammatory cytokines, such as IFN-gamma [29]. In inflammatory myopathy, CD8 T cells destroy non-necrotic muscle fibers, showing a response mediated by HLA class I restricted cytotoxic T cells against surface antigens expressed in muscle fibers. Marzuillo et al. [30] reported that muscle fibers, in an inflamed environment, co-express HLA-G, raising the intriguing possibility that HLA-G protects muscle fibers from injury mediated by NK cells [31, 32]. Locally accumulated inflammatory cells produce a multitude of pro-inflammatory cytokines, including TNF-alpha and IFN-gamma. As these cytokines are known to induce HLA expression in many cell types, it seems likely that HLA-G expression, seen in inflammatory myopathies, at least in part, results from stimulation by locally produced cytokines after injury [33].

Insufficient physical exercise is related to the development of several diseases and metabolic changes, such as decreased insulin sensitivity, changes in lipid metabolism, increased visceral adiposity, decreased lean body mass and loss of muscle strength, resulting in low-grade chronic inflammation [34]. On the other hand, muscle damage, resulting from physical exercise, may initially result in a condition of acute inflammation with an increase in the production of myocins and activation of immune system cells to the site of muscle damage in order to initiate the tissue remodeling and repair process [35]. Noteworthy, it has been shown that systematic physical training can lead to a local and systemic anti-inflammatory state that enables tissue regeneration and adaptation and, at the same time, protects the organism against the development of chronic inflammatory pathologies [36]. In other words, an increase in the pro-inflammatory organic condition, caused by the stimulation of acute exercise, would be counterbalanced by the chronic anti-inflammatory environment, caused by systematic exercise, which would restrict the magnitude and duration of the inflammation. Thus, the beneficial effects of physical training on the modulation of inflammation depend on the quality and quantity of stimuli, which are directly related to the rest time between stimuli [37]. Thus, transversal and longitudinal data suggest that people who perform regular exercises of moderate intensity maintain a protective state [28, 38]. However, intense and prolonged exercise has been associated with greater morbidity and mortality [4, 39]. These findings gave rise to the "J"-shaped model, related the relationship between the exercise dose and the risk of infection. This model reports that moderate exercise may lower the risk of upper respiratory infection, whereas excessive exercise may increase the risk. Indeed, greater risk for upper respiratory infection is observed in over-trained compared with well-trained athletes [40].

Finally, our results present important evidence related to the immunological profile in bodybuilders, associating the decreased body fat to an increased plasma sHLA-G levels, indicating that, at least in part, HLA-G may contribute to the immunosuppressive state of athletes undergoing intense and prolonged physical training. Indeed, bodybuilding performed with extreme training to optimize lean mass and reduce the rate of body fat [31] may be associated with immunosuppression process [41], which has been attributed to exhaustive and prolonged exercises [32, 42, 43]. This cross-sectional study presents data obtained only at the time of championship, and long-term follow-up studies evaluating the role of diet, supplements and the role of the innate and adaptive immune response are needed to clarify the influence of intense physical exercise on the immune function.
